# Supplementary material for: Strike a Pose: Relationships Between Infants’ Motor Development and Visuospatial Representations of Bodies
Source: Behav Sci (Basel). 2025 Jul 28;15(8):1021. doi: 10.3390/bs15081021 (PMC12383127; doi:10.3390/bs15081021)

Supplementary Materials

Table S1

Study 2B Fixed Effect Omnibus Tests for Linear Mixed Effects Model Comparing Novelty Preferences Across Age, Condition and Test Trial and Relationships with Gross and Fine Motor

	F	Num *df*	Den *df*	*p*
Age	0.02	1	357.00	.879
Condition	0.26	3	357.00	.858
Gross	1.59	1	357.00	.208
Fine	0.08	1	357.00	.779
Test Trial	1.54	1	357.00	.216
Age ✻ Condition	0.66	3	357.00	.576
Age ✻ Gross	0.99	1	357.00	.321
Age ✻ Fine	0.58	1	357.00	.448
Age ✻ Test Trial	2.80	1	357.00	.095
Condition ✻ Test Trial	2.20	3	357.00	.087
Age ✻ Condition ✻ Gross	0.35	6	357.00	.909
Age ✻ Condition ✻ Fine	0.24	6	357.00	.964
**Age ✻ Condition ✻ Test Trial**	**5.00**	**3**	**357.00**	**.002**

Table S2

Study 2B Novelty Preferences Fixed Effects Parameter Estimates for the Age, Condition, Test Trial Comparisons and Relationships with Fine and Gross Motor

Names	Effect	Estimate	*SE*	95% Confidence Interval Lower	Upper	*df*	*t*	*p*
(Intercept)	(Intercept)	0.50	0.01	0.48	0.52	357.00	51.52	< .001
Age	9 - 7	0.00	0.02	-0.04	0.04	357.00	0.15	.879
Condition1	HLI - HLU	-0.01	0.03	-0.06	0.04	357.00	-0.33	.745
Condition2	HLU - WFI	-0.01	0.03	-0.06	0.04	357.00	-0.38	.701
Condition3	WFI - WFU	0.02	0.03	-0.03	0.08	357.00	0.79	.432
Gross	Gross	-0.00	0.00	-0.00	0.00	357.00	-1.26	.208
Fine	Fine	0.00	0.00	-0.00	0.00	357.00	0.28	.779
Test Trial	2 - 1	-0.02	0.02	-0.06	0.01	357.00	-1.24	.216
Age ✻ Condition1	9 - 7 ✻ HLI - HLU	0.05	0.05	-0.06	0.15	357.00	0.87	.385
Age ✻ Condition2	9 - 7 ✻ HLU - WFI	-0.07	0.05	-0.18	0.04	357.00	-1.28	.202
Age ✻ Condition3	9 - 7 ✻ WFI - WFU	0.01	0.06	-0.10	0.12	357.00	0.15	.885
Age ✻ Gross	9 - 7 ✻ Gross	0.00	0.00	-0.00	0.00	357.00	1.26	.210
Age ✻ Fine	9 - 7 ✻ Fine	-0.00	0.00	-0.00	0.00	357.00	-0.87	.385
Age ✻ Test Trial	9 - 7 ✻ 2 - 1	0.06	0.04	-0.01	0.14	357.00	1.67	.095
Condition1 ✻ Test Trial	HLI - HLU ✻ 2 - 1	0.01	0.05	-0.10	0.11	357.00	0.15	.878
Condition2 ✻ Test Trial	HLU - WFI ✻ 2 - 1	-0.05	0.05	-0.16	0.05	357.00	-0.98	.327
**Condition3 ✻ Test Trial**	**WFI - WFU ✻ 2 - 1**	**0.14**	**0.05**	**0.03**	**0.24**	**357.00**	**2.51**	**.012**
7m ✻ Cond1 ✻ Gross	7m ✻ HLI - HLU ✻ Gross	-0.00	0.00	-0.01	0.00	357.00	-1.18	.239
9m ✻ Cond1 ✻ Gross	9m ✻ HLI - HLU ✻ Gross	0.00	0.00	-0.00	0.00	357.00	0.08	.934
7m ✻ Cond2 ✻ Gross	7m ✻ HLU - WFI ✻ Gross	0.00	0.00	-0.00	0.01	357.00	0.73	.464
9m ✻ Cond2 ✻ Gross	9m ✻ HLU - WFI ✻ Gross	-0.00	0.00	-0.01	0.00	357.00	-0.40	.688
7m ✻ Cond3 ✻ Gross	7m ✻ WFI - WFU ✻ Gross	0.00	0.00	-0.01	0.01	357.00	0.27	.784
9m ✻ Cond3 ✻ Gross	9m ✻ WFI - WFU ✻ Gross	-0.00	0.00	-0.00	0.00	357.00	-0.20	.845
7m ✻ Cond1 ✻ Fine	7m ✻ HLI - HLU ✻ Fine	0.00	0.00	-0.01	0.01	357.00	0.25	.802
9m ✻ Cond1 ✻ Fine	9m ✻ HLI - HLU ✻ Fine	-0.00	0.00	-0.01	0.00	357.00	-0.92	.356

Names	Effect	Estimate	*SE*	95% Confidence Interval Lower	Upper	*df*	*t*	*p*
7m ✻ Cond2 ✻ Fine	7m ✻ HLU - WFI ✻ Fine	-0.00	0.00	-0.01	0.00	357.00	-0.57	.566
9m ✻ Cond2 ✻ Fine	9m ✻ HLU - WFI ✻ Fine	0.00	0.00	-0.00	0.01	357.00	0.84	.400
7m ✻ Cond3 ✻ Fine	7m ✻ WFI - WFU ✻ Fine	0.00	0.00	-0.00	0.01	357.00	0.41	.684
9m ✻ Cond3 ✻ Fine	9m ✻ WFI - WFU ✻ Fine	-0.00	0.00	-0.01	0.01	357.00	-0.17	.865
**Age ✻ Cond1 ✻ Test Trial**	**9 - 7 ✻ HLI - HLU ✻ 2 - 1**	**0.30**	**0.11**	**0.09**	**0.51**	**357.00**	**2.81**	**.005**
**Age ✻ Cond2 ✻ Test Trial**	**9 - 7 ✻ HLU - WFI ✻ 2 - 1**	**-0.38**	**0.11**	**-0.59**	**-0.17**	**357.00**	**-3.54**	**< .001**
**Age ✻ Cond3 ✻ Test Trial**	**9 - 7 ✻ WFI - WFU ✻ 2 - 1**	**0.24**	**0.11**	**0.03**	**0.46**	**357.00**	**2.22**	**.027**

Note. HLI = Headless Inverted; HLU = Headless Upright; WFI = Whole Figure Inverted; WFU = Whole Figure Upright

Table S3

Study 2B Novelty Preferences Follow-up Simple Effects for Age, Condition, and Test Trial

Moderator levels					95% Confidence Interval				
Age	Test_Trial	contrast	Estimate	*SE*	Lower	Upper	*df*	*t*	*p*
7	1	HLI - HLU	0.04	0.05	-0.06	0.14	357.00	0.74	.462
		WFI - WFU	0.01	0.05	-0.09	0.11	357.00	0.19	.851
	**2**	**HLI - HLU**	**-0.10**	**0.05**	**-0.21**	**-0.00**	**357.00**	**-2.00**	**.047***
		WFI - WFU	0.03	0.05	-0.08	0.13	357.00	0.47	.636
9	1	HLI - HLU	-0.06	0.06	-0.17	0.04	357.00	-1.16	.247
		WFI - WFU	-0.10	0.06	-0.21	0.01	357.00	-1.85	.065
	2	HLI - HLU	0.09	0.06	-0.02	0.21	357.00	1.66	.098
		**WFI - WFU**	**0.16**	**0.06**	**0.04**	**0.27**	**357.00**	**2.72**	**.007**

Note. HLI = Headless Inverted; HLU = Headless Upright; WFI = Whole Figure Inverted; WFU = Whole Figure Upright; *non-significant with Benjamini-Hochberg FDR adjustments.

Table S4

Test Trial One: Relationship Between Novelty Preferences and Low, Medium, and High Levels of Gross Motor in Each Orientation Comparison

Moderator levels					95% Confidence Interval				
Age	ASQ Gross	contrast	Estimate	*SE*	Lower	Upper	*df*	*t*	*p*
7	Mean -1 *SD* (≤30)	HLI - HLU	0.03	0.09	-0.14	0.21	182.00	0.39	.699
		WFI - WFU	0.03	0.09	-0.15	0.22	182.00	0.35	.727
	Mean (31 to 52)	HLI - HLU	0.03	0.05	-0.06	0.13	182.00	0.66	.508
		WFI - WFU	0.01	0.05	-0.09	0.11	182.00	0.26	.798
	Mean +1 *SD* (>53)	HLI - HLU	0.03	0.07	-0.11	0.17	182.00	0.42	.672
		WFI - WFU	-0.01	0.07	-0.14	0.13	182.00	-0.09	.925
9	Mean -1 SD (≤18)	HLI - HLU	-0.06	0.06	-0.18	0.06	182.00	-0.99	.325
		WFI - WFU	-0.10	0.06	-0.22	0.03	182.00	-1.57	.119
	Mean (19 to 52)	HLI - HLU	-0.07	0.05	-0.17	0.03	182.00	-1.33	.185
		WFI - WFU	-0.10	0.05	-0.20	0.00	182.00	-1.95	.053
	Mean +1 *SD* (≥53)	HLI - HLU	-0.08	0.07	-0.23	0.07	182.00	-1.07	.288
		WFI - WFU	-0.10	0.07	-0.25	0.04	182.00	-1.44	.153

Note. WFU = Whole Figure Upright; WFI = Whole Figure Inverted; HLU = Headless Upright; HLI = Headless Inverted

Table S5

Test Trial Two: Relationship Between Novelty Preferences and Low, Medium, and High Levels of Gross Motor in Each Orientation Comparison

Moderator levels					95% Confidence Interval				
Age	ASQ Gross	contrast	Estimate	*SE*	Lower	Upper	*df*	*t*	*p*
7	Mean -1 *SD* (≤30)	HLI - HLU	0.00	0.09	-0.18	0.19	175.00	0.05	.961
		WFI - WFU	-0.03	0.10	-0.22	0.17	175.00	-0.26	.792
	Mean (31 to 52)	HLI - HLU	-0.10	0.05	-0.20	0.01	175.00	-1.81	.072
		WFI - WFU	0.03	0.06	-0.08	0.14	175.00	0.47	.636
	**Mean +1 *SD*** (≥53)	**HLI - HLU**	**-0.20**	**0.08**	**-0.35**	**-0.04**	**175.00**	**-2.48**	**.014**
		WFI - WFU	0.08	0.08	-0.08	0.24	175.00	0.96	.338
9	**Mean -1 *SD*** (≤18)	HLI - HLU	0.09	0.07	-0.05	0.22	175.00	1.27	.205
		**WFI - WFU**	**0.17**	**0.07**	**0.02**	**0.31**	**175.00**	**2.30**	**.023**
	**Mean** (19 to 52)	HLI - HLU	0.10	0.06	-0.01	0.22	175.00	1.75	.082
		**WFI - WFU**	**0.16**	**0.06**	**0.04**	**0.27**	**175.00**	**2.68**	**.008**
	Mean +1 SD (≥53)	HLI - HLU	0.12	0.08	-0.04	0.28	175.00	1.44	.153
		WFI - WFU	0.14	0.08	-0.01	0.30	175.00	1.82	.071

Note. WFU = Whole Figure Upright; WFI = Whole Figure Inverted; HLU = Headless Upright; HLI = Headless Inverted

Table S6

Test Trial One: Relationship Between Novelty Preferences and Low, Medium, and High Levels of Fine Motor in Each Orientation Comparison

Moderator levels					95% Confidence Interval				
Age	ASQ Fine	contrast	Estimate	*SE*	Lower	Upper	*df*	*t*	*p*
7	Mean -1 *SD* (≤39)	HLI - HLU	0.08	0.07	-0.06	0.22	182.00	1.14	.254
		WFI - WFU	0.05	0.06	-0.08	0.17	182.00	0.72	.471
	Mean (40 to 59)	HLI - HLU	0.03	0.05	-0.06	0.13	182.00	0.66	.508
		WFI - WFU	0.01	0.05	-0.09	0.11	182.00	0.26	.798
	Mean +1 SD (60)	HLI - HLU	-0.02	0.07	-0.15	0.12	182.00	-0.23	.819
		WFI - WFU	-0.02	0.07	-0.15	0.11	182.00	-0.30	.762
9	**Mean -1 *SD*** (≤39)	HLI - HLU	-0.08	0.08	-0.23	0.07	182.00	-1.02	.309
		**WFI - WFU**	**-0.17**	**0.08**	**-0.33**	**-0.02**	**182.00**	**-2.20**	**.029***
	Mean (40 to 59)	HLI - HLU	-0.07	0.05	-0.17	0.03	182.00	-1.33	.185
		WFI - WFU	-0.10	0.05	-0.20	0.00	182.00	-1.95	.053
	Mean +1 *SD* (60)	HLI - HLU	-0.06	0.08	-0.21	0.09	182.00	-0.79	.431
		WFI - WFU	-0.03	0.08	-0.18	0.12	182.00	-0.38	.703

Note. WFU = Whole Figure Upright; WFI = Whole Figure Inverted; HLU = Headless Upright; HLI = Headless Inverted; *non-significant with Benjamini-Hochberg FDR adjustments.

Table S7

Test Trial Two: Relationship Between Novelty Preferences and Low, Medium, and High Levels of Fine Motor in Each Orientation Comparison

Moderator levels					95% Confidence Interval				
Age	ASQ_Fine	contrast	Estimate	*SE*	Lower	Upper	*df*	*t*	*p*
**7**	**Mean -1 *SD*** (≤39)	**HLI - HLU**	**-0.16**	**0.07**	**-0.30**	**-0.01**	**175.00**	**-2.17**	**.032***
		WFI - WFU	-0.04	0.07	-0.18	0.11	175.00	-0.51	.610
	Mean (40 to 59)	HLI - HLU	-0.10	0.05	-0.20	0.01	175.00	-1.81	.072
		WFI - WFU	0.03	0.06	-0.08	0.14	175.00	0.47	.636
	Mean +1 *SD* (60)	HLI - HLU	-0.04	0.07	-0.18	0.11	175.00	-0.50	.619
		WFI - WFU	0.09	0.08	-0.06	0.24	175.00	1.17	.245
**9**	**Mean -1 *SD*** (≤39)	**HLI - HLU**	**0.19**	**0.09**	**0.02**	**0.37**	**175.00**	**2.17**	**.032***
		**WFI - WFU**	**0.25**	**0.09**	**0.07**	**0.42**	**175.00**	**2.76**	**.006**
	**Mean** (40 to 59)	HLI - HLU	0.10	0.06	-0.01	0.22	175.00	1.75	.082
		**WFI - WFU**	**0.16**	**0.06**	**0.04**	**0.27**	**175.00**	**2.68**	**.008**
	Mean +1 *SD* (60)	HLI - HLU	0.01	0.08	-0.15	0.17	175.00	0.11	.914
		WFI - WFU	0.06	0.09	-0.11	0.24	175.00	0.73	.464

Note. WFU = Whole Figure Upright; WFI = Whole Figure Inverted; HLU = Headless Upright; HLI = Headless Inverted; *non-significant with Benjamini-Hochberg FDR adjustments.

Study 3 – Bodies, Heads, and Feet Looking Proportions Across Conditions and Studies

The amount of focus infants had on the bodies, heads, and feet were assessed to determine whether attention to the bodies or upper and lower extremities of the figures were influential in the ability to discriminate the postures. This was compared across conditions and studies to assess effects of stimulus complexity, face direction and trial duration on infants' focus on heads, bodies, and feet. Age was collapsed across the 8-second similar-sized study (Study 2B) as there was no significant difference in age for looking at the bodies in this study ($p = .781$).

Body Looking Time Proportions

A LMM analysis was performed comparing the body looking time proportions across the conditions (repeated factors: whole figure upright vs. whole figure inverted, headless upright vs. headless inverted), and studies (5-second original, about-facing, 5-second similarly-varied, 8-second similarly-varied postures) and an interaction between condition and study. The random effect of participant was significant, $LRT(1) = 35.48$, $p < .001$ (random intercept variance = 0.01 $SD = 0.08$), and the intra-class correlation (*ICC*) was 0.13 suggesting a small amount of variability across participants. The main effect of condition was significant, $F(3,788.77) = 321.62$, $p < .001$. There was significantly greater looking at the bodies in the headless upright ($M = 0.94$, $SE = 0.02$) than the headless inverted condition ($M = 0.67$, $SE = 0.02$), but for the whole figure conditions it was the opposite with significantly greater looking at bodies in the inverted ($M = 0.56$, $SE = 0.02$) than the upright condition ($M = 0.30$, $SE = 0.02$) (see Table S8 for all fixed effects parameter estimates). Post-hoc Bonferroni-corrected comparisons also revealed that all conditions differed to each other in body looking time proportions with the significantly greatest looking in the upright headless condition, followed by the inverted headless, inverted whole figures and the shortest duration was in the upright whole figure condition (all *p*s < .001, see Table S9; see also Figure S1). Therefore, the presence of heads even when inverted, detract attention from bodies. There was a main effect of study, $F(3,126.50) = 3.78$, $p = .012$. This was

explained by significantly lower looking at bodies in the 8-second similar-sized study (M = 0.57, SE = 0.02) compared to the about-facing study (M = 0.66, SE = 0.02) (see Table S8 and Figure S1). The interaction between study and condition was significant, $F(9,788.12)$ = 2.01, p = .036. This was due to greater looking at the inverted compared to the upright whole figure bodies being more pronounced in the 8-second similar-sized study (0.25) than in the about-facing study (0.16, see Table S8 and Figure S1). This was also the case for the 5-second similar-sized study (WFI – WFU = 0.58) compared to the about-facing study (see Table S8 and Figure S1). A follow-up simple effects test also revealed that for the whole figure condition, there was significantly greater looking at the bodies in the about-facing study compared to all other studies (5-second original: p = .011; 5-second similar-sized: p <.002; 8-second similar-sized: p < .001, see Figure S1, and Table S10). These effects remained significant following adjustments for multiple tests (Benjamini-Hochberg FDR adjustments for multiple comparisons across 3 comparisons, α<.0167). Post-hoc Bonferroni-corrected comparisons revealed that the looking proportions to bodies were non-significant between all remaining studies (all ps > .05).

Table S8

Body Looking Time Proportion Fixed Effects Across Conditions and Studies

				95% Confidence Interval				
Names	Effect	Estimate	*SE*	Lower	Upper	*df*	*t*	*p*
(Intercept)	(Intercept)	0.62	0.01	0.60	0.64	126.98	57.33	< .001
**Condition**	**HLI - HLU**	**-0.28**	**0.02**	**-0.32**	**-0.24**	**792.02**	**-12.97**	**< .001**
**Condition**	**HLU - WFI**	**0.38**	**0.02**	**0.34**	**0.42**	**786.00**	**18.20**	**< .001**
**Condition**	**WFI - WFU**	**0.26**	**0.02**	**0.22**	**0.31**	**785.16**	**12.57**	**< .001**
**Study**	**Eight_S_Similar - AboutFacing**	**-0.09**	**0.03**	**-0.14**	**-0.03**	**122.61**	**-3.20**	**.002**
Study	Original_5s_7m - AboutFacing	-0.03	0.03	-0.10	0.03	127.74	-1.01	.312
Study	Similar_5s_7m - AboutFacing	-0.05	0.03	-0.12	0.01	125.67	-1.69	.094
Condition ✻ Study	HLI - HLU ✻ Eight_S_Similar - AboutFacing	-0.01	0.05	-0.11	0.10	781.74	-0.14	.890
Condition ✻ Study	HLU - WFI ✻ Eight_S_Similar - AboutFacing	0.02	0.05	-0.09	0.13	788.24	0.37	.708
**Condition ✻ Study**	**WFI - WFU ✻ Eight_S_Similar - AboutFacing**	**0.11**	**0.05**	**0.00**	**0.22**	**782.93**	**1.97**	**.049**
Condition ✻ Study	HLI - HLU ✻ Original_5s_7m - AboutFacing	0.05	0.07	-0.08	0.17	793.32	0.70	.482
Condition ✻ Study	HLU - WFI ✻ Original_5s_7m - AboutFacing	-0.01	0.06	-0.13	0.12	785.49	-0.12	.905
Condition ✻ Study	WFI - WFU ✻ Original_5s_7m - AboutFacing	0.11	0.07	-0.02	0.23	788.33	1.62	.105
Condition ✻ Study	HLI - HLU ✻ Similar_5s_7m - AboutFacing	0.11	0.06	-0.02	0.23	787.51	1.65	.099
Condition ✻ Study	HLU - WFI ✻ Similar_5s_7m - AboutFacing	-0.06	0.06	-0.19	0.06	790.56	-0.95	.341
**Condition ✻ Study**	**WFI - WFU ✻ Similar_5s_7m - AboutFacing**	**0.19**	**0.06**	**0.06**	**0.31**	**780.34**	**2.94**	**.003**

Note. WFU = Whole Figure Upright; WFI = Whole Figure Inverted; HLU = Headless Upright; HLI = Headless Inverted

Table S9

Study 3 Body Looking Time Proportion Comparisons Between Each Condition

			Difference	*SE*	*t*	*df*	*p*
HLI	-	HLU	-0.28	0.02	-12.86	804.61	< .001
HLI	-	WFI	0.10	0.02	4.84	805.37	< .001
HLI	-	WFU	0.37	0.02	17.06	800.22	< .001
HLU	-	WFI	0.38	0.02	18.06	798.46	< .001
HLU	-	WFU	0.65	0.02	30.42	801.88	< .001
WFI	-	WFU	0.26	0.02	12.47	797.62	< .001

Note. WFU = Whole Figure Upright; WFI = Whole Figure Inverted; HLU = Headless Upright; HLI = Headless Inverted

Table S10

Study 3 Body Looking Time Proportions Across Studies in Each Condition

Moderator levels				95% Confidence Interval				
Condition	contrast	Estimate	*SE*	Lower	Upper	*df*	*t*	*p*
HLI	8s-Similar – AboutFacing	-0.05	0.04	-0.13	0.04	561.98	-1.12	.262
	Original_5s - AboutFacing	0.03	0.05	-0.07	0.13	583.36	0.62	.537
	Similar_5s - AboutFacing	0.02	0.05	-0.08	0.12	586.87	0.39	.694
HLU	8s-Similar - AboutFacing	-0.04	0.04	-0.13	0.04	530.36	-1.07	.285
	Original_5s - AboutFacing	-0.02	0.05	-0.12	0.08	558.76	-0.36	.719
	Similar_5s - AboutFacing	-0.06	0.05	-0.16	0.04	563.94	-1.14	.253
WFI	8s-Similar - AboutFacing	-0.05	0.04	-0.14	0.03	569.11	-1.18	.237
	Original_5s - AboutFacing	-0.00	0.05	-0.10	0.10	565.16	-0.05	.963
	Similar_5s - AboutFacing	-0.04	0.05	-0.14	0.06	567.49	-0.74	.459
**WFU**	**8s-Similar - AboutFacing**	**-0.15**	**0.04**	**-0.23**	**-0.06**	**561.36**	**-3.45**	**< .001**
	**Original_5s - AboutFacing**	**-0.13**	**0.05**	**-0.23**	**-0.03**	**581.29**	**-2.54**	**.011**
	**Similar_5s - AboutFacing**	**-0.15**	**0.05**	**-0.25**	**-0.05**	**552.48**	**-3.04**	**.002**

Figure S1

Body Looking Time Proportions in each Condition and Study

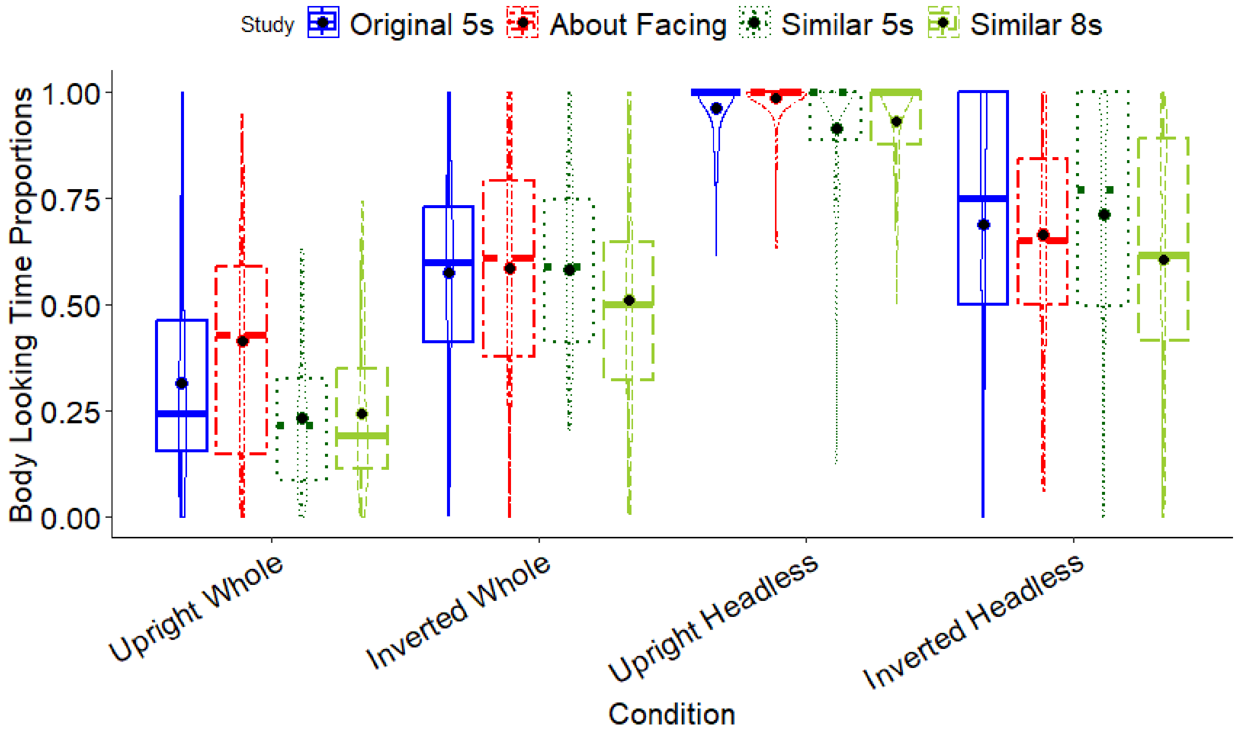

Head Looking Time Proportions

A comparison of the head looking time proportions was performed across the conditions (repeated factors: whole figure upright vs. whole figure reinverted), the studies (5-second original, about-facing, 5-second similar-sized, 8-second similar-sized), and the interaction between conditions and studies. The headless conditions were excluded from this analysis. The random effect of participant was significant, $LRT(1) = 24.98$, $p < .001$ (random intercept variance = 0.01 $SD = 0.10$), and the intra-class correlation (ICC) was 0.22 suggesting small-to-moderate variability across participants. There was a main effect of condition, $F(1,344.32) = 770.72$, $p < .001$, with significantly greater looking at the heads in the upright ($M = 0.69$, $SE = 0.02$) than the inverted condition ($M = 0.15$, $SE = 0.02$). There was also a main effect of study, $F(3,125.98) = 4.99$, $p = .003$, with significantly lower looking at the heads in the about-facing study ($M = 0.33$, $SE = 0.04$) compared to all other studies (original 5s: $M = 0.42$, $SE = 0.03$; similar 5s: $M = 0.46$, $SE = 0.03$; similar 8s: $M = 0.46$, $SE = 0.02$; see Table S10 and Figure S2). The interaction between condition and study was non-significant ($F(3,344.13) = 1.95$, $p = .121$), but looking at the upright compared to the inverted whole figure heads was more pronounced in the 5-second similar-sized study (0.62) than in the about-facing study (0.48, see Table S11 and Figure S2). A follow-up simple effects test also revealed that for the upright stimuli the difference in looking at heads between the about-facing study and the other studies was significant (all *p*s < .05, see Table S12). There was also significantly greater looking at heads of the inverted stimuli in the similar 8s study compared to the about-facing, but this was no longer significant following adjustments for multiple tests (Benjamini-Hochberg FDR adjustments for multiple comparisons across 3 comparisons, $\alpha<.0167$).

Table S11

Head Looking Time Proportion Fixed Effects Across Conditions and Studies

				95% Confidence Interval				
Names	Effect	Estimate	*SE*	Lower	Upper	*df*	*t*	*p*
(Intercept)	(Intercept)	0.42	0.01	0.39	0.44	126.33	30.59	< .001
**Condition1**	**WFI - WFU**	**-0.54**	**0.02**	**-0.58**	**-0.50**	**344.32**	**-27.76**	**< .001**
**Study1**	**Eight_S_Similar - AboutFacing**	**0.13**	**0.04**	**0.06**	**0.20**	**125.91**	**3.67**	**< .001**
**Study2**	**Original_5s_7m - AboutFacing**	**0.10**	**0.04**	**0.01**	**0.18**	**129.06**	**2.29**	**.024**
**Study3**	**Similar_5s_7m - AboutFacing**	**0.13**	**0.04**	**0.05**	**0.21**	**125.07**	**3.17**	**.002**
Condition1 ✻ Study1	WFI - WFU ✻ Eight_S_Similar - AboutFacing	-0.06	0.05	-0.16	0.04	343.05	-1.12	.266
Condition1 ✻ Study2	WFI - WFU ✻ Original_5s_7m - AboutFacing	-0.03	0.06	-0.15	0.08	347.36	-0.58	.561
**Condition1 ✻ Study3**	**WFI - WFU ✻ Similar_5s_7m - AboutFacing**	**-0.13**	**0.06**	**-0.25**	**-0.02**	**340.23**	**-2.32**	**.021**

Table S12

Study 3 Head Looking Time Proportions Across Studies in Each Condition

Moderator levels				95% Confidence Interval				
Condition	contrast	Estimate	*SE*	Lower	Upper	*df*	*t*	*p*
**WFI**	**Eight_S_Similar - AboutFacing**	**0.10**	**0.04**	**0.01**	**0.18**	**257.31**	**2.18**	**.030***
	Original_5s_7m - AboutFacing	0.09	0.05	-0.02	0.19	257.17	1.66	.098
	Similar_5s_7m - AboutFacing	0.09	0.05	-0.01	0.19	258.04	1.76	.080
**WFU**	**Eight_S_Similar - AboutFacing**	**0.13**	**0.04**	**0.05**	**0.22**	**255.32**	**3.06**	**.002**
	**Original_5s_7m - AboutFacing**	**0.13**	**0.05**	**0.02**	**0.23**	**266.48**	**2.44**	**.015**
	**Similar_5s_7m - AboutFacing**	**0.16**	**0.05**	**0.06**	**0.26**	**252.18**	**3.23**	**.001**

*non-significant with Benjamini-Hochberg FDR adjustments.

Figure S2

Head Looking Time Proportions in each Condition and Study

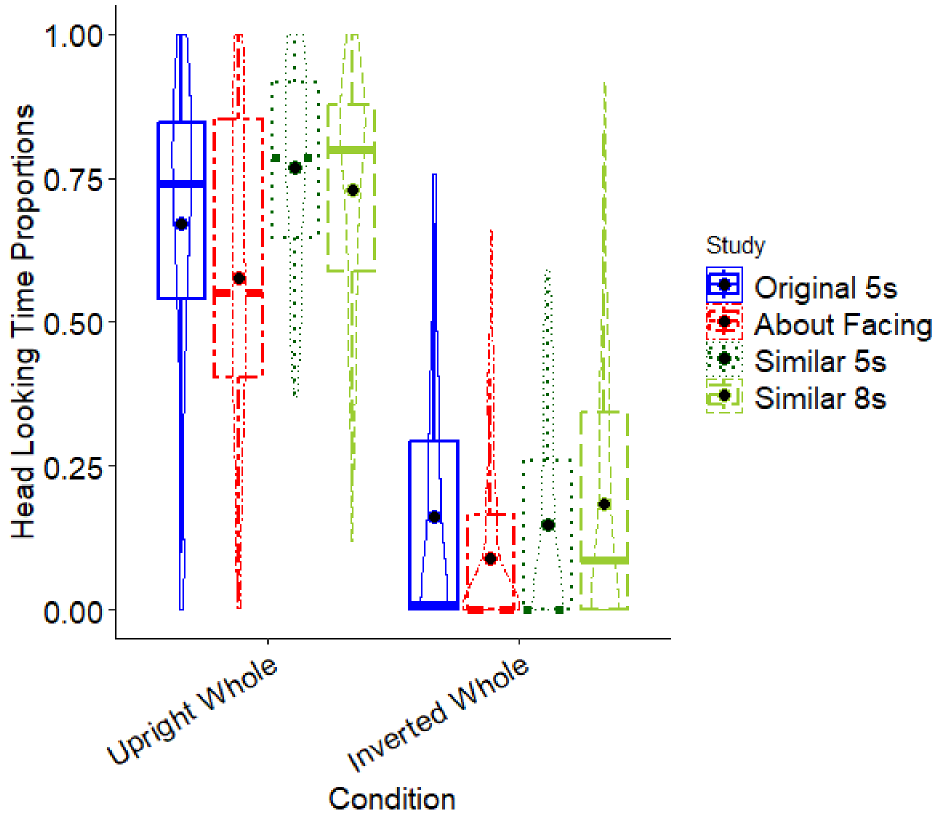

Feet Looking Time Proportions

A comparison between feet looking time proportions was performed across the conditions (repeated factors: whole figure upright vs. whole figure inverted, headless upright vs. headless inverted), the studies (5-second original, about-facing, 5-second similar-sized, 8-second similar-sized), and the interaction between conditions and studies. The random effect of participant was significant, *LRT*(1) = 27.05, *p* < .001 (random intercept variance = 0.001, *SD* = 0.07), and the intra-class correlation (*ICC*) was 0.11 suggesting a small amount of variability across participants. The main effect of condition was significant, *F*(3,791.50) = 145.52, *p* < .001. There was significantly greater looking at the feet in the headless inverted (*M* = 0.33, *SE* = 0.02) than the headless upright condition (*M* = 0.03, *SE* = 0.02), and in the whole figure inverted (*M* = 0.29, *SE* = 0.02) compared to the whole figure upright condition (*M* = 0.02, *SE* = 0.02; see Table S13 and Figure S3). The main effect of study, *F*(3,127.97) =

1.65, p = .182, and the condition by study interaction were non-significant, F(9,790.81) = 0.80, p = .605.

Table S13

Feet Looking Time Proportion Fixed Effects Across Conditions and Studies

				95% Confidence Interval				
Names	Effect	Estimate	*SE*	Lower	Upper	*df*	*t*	*p*
(Intercept)	(Intercept)	0.17	0.01	0.15	0.19	128.50	17.71	< .001
**Condition1**	**HLI - HLU**	**0.30**	**0.02**	**0.26**	**0.34**	**794.93**	**15.12**	**< .001**
**Condition2**	**HLU - WFI**	**-0.26**	**0.02**	**-0.30**	**-0.22**	**788.61**	**-13.36**	**< .001**
**Condition3**	**WFI - WFU**	**0.28**	**0.02**	**0.24**	**0.31**	**787.62**	**14.29**	**< .001**
Study1	8s Similar - AboutFacing	0.03	0.02	-0.02	0.07	123.77	1.11	.271
Study2	Original_5s_7m - AboutFacing	-0.02	0.03	-0.07	0.04	129.38	-0.59	.555
Study3	Similar_5s_7m - AboutFacing	-0.02	0.03	-0.07	0.04	127.08	-0.57	.569
Condition1 ✻ Study1	HLI - HLU ✻ 8s Similar - AboutFacing	0.00	0.05	-0.09	0.10	784.14	0.06	.955
Condition2 ✻ Study1	HLU - WFI ✻ 8s Similar - AboutFacing	0.09	0.05	-0.01	0.18	790.88	1.73	.085
Condition3 ✻ Study1	WFI - WFU ✻ Eight_S_Similar - AboutFacing	-0.05	0.05	-0.15	0.05	785.32	-0.97	.334
Condition1 ✻ Study2	HLI - HLU ✻ Original_5s_7m - AboutFacing	-0.04	0.06	-0.15	0.08	796.32	-0.60	.551
Condition2 ✻ Study2	HLU - WFI ✻ Original_5s_7m - AboutFacing	0.07	0.06	-0.05	0.19	788.08	1.19	.233
Condition3 ✻ Study2	WFI - WFU ✻ Original_5s_7m - AboutFacing	-0.06	0.06	-0.18	0.05	790.94	-1.09	.278
Condition1 ✻ Study3	HLI - HLU ✻ Similar_5s_7m - AboutFacing	-0.07	0.06	-0.19	0.05	790.23	-1.17	.241
Condition2 ✻ Study3	HLU - WFI ✻ Similar_5s_7m - AboutFacing	0.09	0.06	-0.03	0.20	793.44	1.52	.128
Condition3 ✻ Study3	WFI - WFU ✻ Similar_5s_7m - AboutFacing	-0.05	0.06	-0.16	0.07	782.60	-0.84	.401

Note. WFU = Whole Figure Upright; WFI = Whole Figure Inverted; HLU = Headless Upright; HLI = Headless Inverted

Figure S3

Feet Looking Time Proportions in each Condition and Study

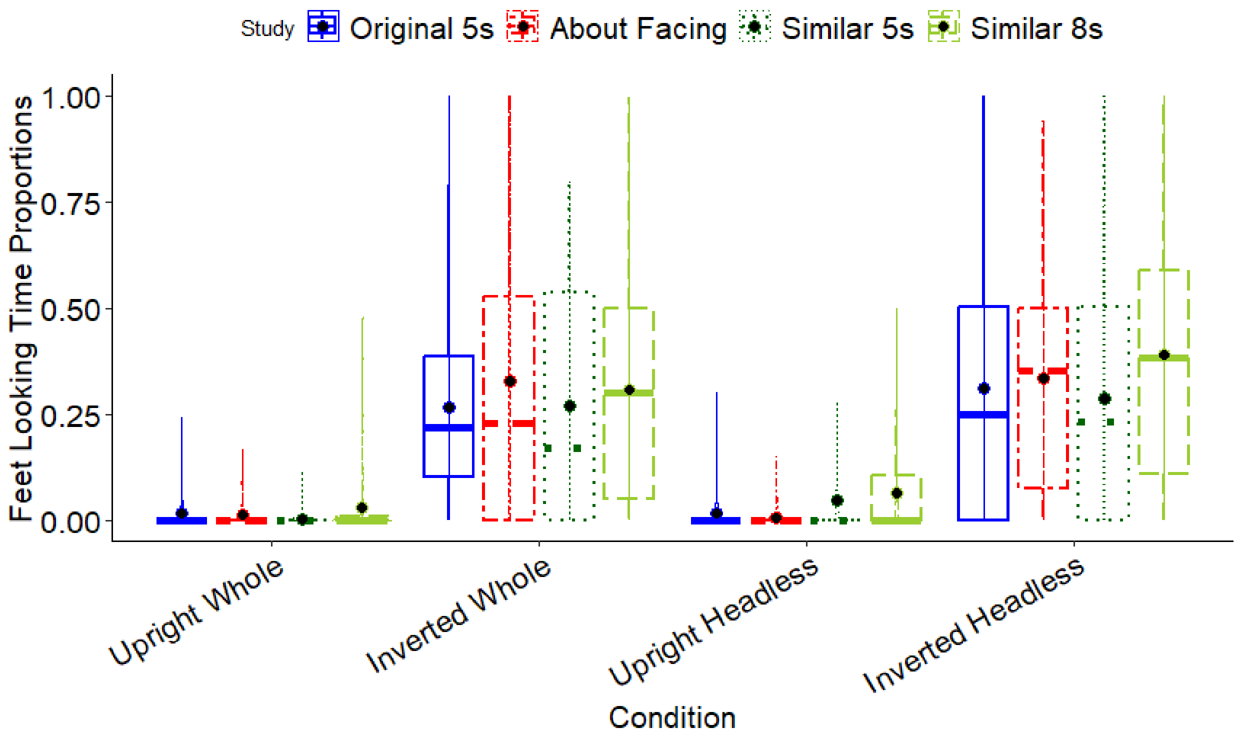

Supplement: Supplementary file 1 [file behavsci-15-01021-s001.zip › behavsci-3532648-supplementary.pdf]
